# The delivery of preventive care to clients of community health services

**DOI:** 10.1186/1472-6963-13-167

**Published:** 2013-05-06

**Authors:** Kathleen M McElwaine, Megan Freund, Elizabeth M Campbell, Jenny Knight, Jennifer A Bowman, Emma L Doherty, Paula M Wye, Luke Wolfenden, Christophe Lecathelinais, Scott McLachlan, John H Wiggers

**Affiliations:** 1 Population Health, Hunter New England Local Health District, Booth Building, Wallsend Health Services, Longworth Avenue, Wallsend, NSW 2287, Australia; 2 The University of Newcastle, University Drive, Callaghan, NSW 2308, Australia; 3 Hunter Medical Research Institute, Clinical Research Centre, Lot 1 Kookaburra Circuit, New Lambton Heights, NSW 2305, Australia; 4 Primary and Community Networks, Hunter New England Local Health District, Tamworth Regional Office, 468-472 Peel Street, Tamworth, NSW 2340, Australia

**Keywords:** Community health services, Delivery of health care, Heath prevention, Health risk behaviors, Health care providers

## Abstract

**Background:**

Smoking, poor nutrition, risky alcohol use, and physical inactivity are the primary behavioral risks for common causes of mortality and morbidity. Evidence and guidelines support routine clinician delivery of preventive care. Limited evidence describes the level delivered in community health settings. The objective was to determine the: prevalence of preventive care provided by community health clinicians; association between client and service characteristics and receipt of care; and acceptability of care. This will assist in informing interventions that facilitate adoption of opportunistic preventive care delivery to all clients.

**Methods:**

In 2009 and 2010 a telephone survey was undertaken of 1284 clients across a network of 56 public community health facilities in one health district in New South Wales, Australia. The survey assessed receipt of preventive care (assessment, brief advice, and referral/follow-up) regarding smoking, inadequate fruit and vegetable consumption, alcohol overconsumption, and physical inactivity; and acceptability of care.

**Results:**

Care was most frequently reported for smoking (assessment: 59.9%, brief advice: 61.7%, and offer of referral to a telephone service: 4.5%) and least frequently for inadequate fruit or vegetable consumption (27.0%, 20.0% and 0.9% respectively). Sixteen percent reported assessment for all risks, 16.2% received brief advice for all risks, and 0.6% were offered a specific referral for all risks. The following were associated with increased care: diabetes services, number of appointments, being male, Aboriginal, unemployed, and socio-economically disadvantaged. Acceptability of preventive care was high (76.0%-95.3%).

**Conclusions:**

Despite strong client support, preventive care was not provided opportunistically to all, and was preferentially provided to select groups. This suggests a need for practice change strategies to enhance preventive care provision to achieve adherence to clinical guidelines.

## Background

The primary behavioral risks for the most common causes of mortality and morbidity in developed countries, including Australia include tobacco smoking, inadequate nutrition, risky alcohol use and physical inactivity [1-7]. Most adults in developed nations (up to 92%) have at least one behavioral chronic disease risk [8-11]. Clinical guidelines that recommend routine, opportunistic clinician delivery of preventive care to all clients regarding multiple risks represent one strategy for reducing this preventable disease burden [12-15]. Such recommendations include assessment, and for those ‘at risk’, the provision of brief advice and referral or follow-up [12-19]. Further, both international and national guidelines [15,18,20] state that such care should be provided across the whole health system as a component of all ‘curative’ visits and interventions, regardless of the age or health status of the client. Review evidence supports the efficacy of preventive care [21-25].

Community health services represent a key clinical setting for the provision of preventive care [26-33] as they have historically had a focus on chronic disease prevention, early intervention and health education [29,31], and are a key provider of primary health care in many countries [26,29,31]. Such services can involve contact with health care providers on multiple occasions for the delivery of specialized, non-acute care [29,30,34]. Despite this being a key setting, the research that has described the prevalence of preventive care delivery in this setting has been limited in terms of a focus on single risks [35-41], and on assessment of risk [34-42] and/or brief advice [35-43] rather than referral/follow-up [34-36,40,41]. Further, most studies have described the prevalence of preventive care in single, or a limited number of facilities [34,37-40,42], rather than across networks or large numbers of facilities. Finally, past studies have most commonly relied on clinician self-report, a measurement approach likely to result in an overestimate of care delivery [44]. Previous studies suggest variable and often sub-optimal levels of preventive care provision, particularly regarding client referral or follow-up [36-38,40,41].

If the intended benefits of preventive care provision in community health settings are to be realized, and interventions for increasing such care delivery are to be effective and appropriately targeted, a greater understanding of both the extent and variability of preventive care delivery is required. A number of client and service characteristics have previously been suggested to be associated with the delivery of preventive care in community health services, including client age (older) [38,39], race (Caucasian) [39], socio-economic status (least disadvantaged) [34], service/provider type (health professionals versus paraprofessionals [41]; wound management, procedures or primary health care versus palliative care [34]; registered nurses versus allied health professionals [45]; prenatal clinics versus pediatric providers [46,47], consultation type (first versus follow-up) [34], and location of practice (rural) [34]. However only one study has considered such associations with each separate element of preventive care provision across all four behavioral risks [45] and none have done this separately for each element of preventive care regarding multiple risks combined.

To address the previously described gaps in knowledge regarding the nature of preventive care provision in community health, a study was undertaken to assess the: prevalence of preventive care (assessment, brief advice, referral/follow-up) provided to community health service clients regarding four behavioral risks; association between client and service characteristics and the delivery of such preventive care; and client acceptability of such care.

## Methods

A cross sectional survey was undertaken over a 12 month period between November 2009 to October 2010 across a network of 56 public community health facilities in one health district in New South Wales (NSW), Australia. The district provides healthcare to approximately 840,000 people residing in urban, regional, rural and remote locations, and employs over 1,400 clinicians.

The data were obtained prior to the implementation of an intervention trial reported previously [48], and approved by the Hunter New England Local Health District (No. 09/06/17/4.03) and the University of Newcastle Human Research Ethics Committees (No. H-2010-1116). The trial involved the sequential rollout of an intervention to three geographical and administratively separate groupings of the 56 community health facilities (Group 1 rural and regional, Group 2 regional, rural and remote, Group 3 urban and rural). Facilities in all three groupings involved a similar mix of community health services (nursing, allied health, child and family, diabetes, aged care, and other service types e.g. rehabilitation, chronic and complex care, women's services, migrant services, renal/dialysis, and regional health service programs), with common policies, standards, governance and performance monitoring processes. Services ineligible for inclusion were: sexual assault, palliative care, aged care assessment teams, dementia, home modification, genetics, and child protection services. Such services were deemed ineligible based upon the advice from the clinical services.

Adult clients were eligible to participate if they: had at least one face to face contact with an eligible service within the prior two weeks; spoke English; were mentally and physically capable of completing the interview (determined at interview, or by clinician/family discretion prior); and were not involved in another community health study.

Each week, over 12 consecutive months, approximately 48 clients from each of the three facility groupings [48] within the health district were randomly selected from client electronic medical records. Selected clients were mailed an information letter and telephoned to determine eligibility and consent. Data were obtained via computer-assisted telephone interviews (CATI) (approximately 25 minutes), and from client electronic medical records.

Client and service characteristic information collected by the CATI included: employment status; Aboriginal or Torres Strait Islander status; marital status; highest level of education achieved; and conditions in the prior two months for which the client needed to take medication or receive medical attention. Client age, gender, country of birth, postcode, service attended (nursing, allied health, child and family, diabetes, aged care, and other service types) and number of visits to the service in the prior 12 months were obtained from client medical records.

Clients were asked to indicate, in the month before seeing the service: their frequency of smoking tobacco products [49]; the number of serves of fruit and of vegetables typically eaten per day [50]; how often they had a drink containing alcohol, the number of standard drinks consumed on a typical drinking day, and how often they consumed four or more standard drinks on any one occasion [51]; and how many days a week they usually did 30 minutes or more of physical activity [52].

Based on national guidelines [53-56], clients were considered to be ‘at risk’ and hence require a preventive health education response if they reported that they: smoked any tobacco products [53]; ate less than two serves of fruit or five serves of vegetables per day [55]; drank more than two standard alcoholic drinks on a typical drinking day or four or more standard drinks on any one occasion [56]; or engaged in less than 30 minutes of physical activity on at least five days of the week [54].

The prevalence of three forms of preventive care was measured. For assessment of behavioral risk status, clients were asked if, during an appointment with the service, the clinician asked: if they smoked any tobacco products; how much fruit and how many vegetables they ate; how much alcohol they drank; and how much physical activity they participated in (yes, no, don’t know).

For provision of brief advice, clients who reported being ‘at risk’ as defined by national guidelines [53-56] were asked whether the clinician advised them: to quit smoking or consider using Nicotine Replacement Therapy; to eat more fruit and/or more vegetables; to reduce the amount of alcohol they consume; or to do more physical activity (yes, no, don’t know).

For the provision of referral/follow-up care, clients who reported smoking were asked if they were offered referral to a free NSW Quitline telephone service. Clients who reported inadequate fruit and/or vegetable intake or physical inactivity were asked if they were offered referral to a free ‘Get Healthy Information and Coaching’ telephone service. Clients with ‘at risk’ alcohol use were asked if they were advised to visit their General Practitioner/Aboriginal Medical Service (GP/AMS). All ‘at risk’ clients were asked if the clinician offered to send a summary of their health risks to their GP/AMS (yes, no, don’t know).

To measure client acceptability of preventive care provision, all clients were asked if it was acceptable for clinicians to assess their health risk behaviors, and for ‘at risk’ clients, if it was acceptable for clinicians to provide brief advice and arrange further support for each risk individually and for all risks combined (strongly disagree, disagree, unsure, agree, strongly agree).

Statistical analyses were undertaken using SAS (version 9.2). Client residential postcodes were used to determine disadvantage (Socio-Economic Indexes for Areas [SEIFA]; cut points: higher NSW half [>991] versus lower NSW half [<=991]) [57] and remoteness (Access/Remoteness Index of Australia [ARIA]; major cities versus regional remote towns) [58]. Descriptive statistics were used to describe client and service characteristics. Comparison of participant and non-participant characteristics was undertaken using chi-square analyses (*p* < .01).

Descriptive statistics were used to examine for each risk separately and for all risks combined, the prevalence of: clients who were ‘at risk’; clinician assessment of risk; clinician brief advice; clinician offer of referral/follow-up; and client acceptability of such care. The analyses were weighted based on the three facility groupings [48] to ensure that the sample was representative of all clients attending the community health services across the health district. The prevalence of care provision for all risks combined was defined as: assessment for all four risks; the provision of brief advice for all of a client’s self-reported risks; and either an offer to send a health risk summary of all their risks to the clients GP/AMS, or an offer of referral/follow-up for all their risks individually (i.e. telephone counseling, or GP/AMS for alcohol).

Associations between client/service characteristics and the provision of each form of care (assessment, brief advice and an offer of referral/follow-up for each individual risk and for all risks combined) were initially analyzed using chi-square analysis. Logistic regression analyses were subsequently undertaken separately for the provision of assessment and brief advice for each of the four individual risks (8 regression models). Regression analyses were not undertaken for referral/follow-up for the four individual risks due to inadequate sample size. Separate chi-square and regression analyses were similarly undertaken to determine such associations with the provision of all three forms of care (assessment, brief advice and referral/follow-up) for all risks combined (3 models). For each of the regression models the analysis was adjusted for the three facility groupings to account for potential cluster effect. Variables with a p-value of 0.20 or less from the chi-square analyses were included in each separate regression model, utilizing a backward stepwise selection process whereby the variable with the highest p value was removed until all predictors in the model had a p value less than .01. Any potential interaction between variables that remained in each of the final models were also examined to ensure the model was sound and that results for each variable could be interpreted independently from other variables [59].

## Results

### Sample

Of 2034 clients randomly selected from the health district’s client electronic medical records, 1767 (86.9%) were eligible. A total of 1284 eligible clients participated in the survey (72.7%) (Table 1) (Facility grouping 1: n = 427, 33.3%; facility grouping 2: n = 408, 31.8%; facility grouping 3: n = 449, 35.0%). This sample size of 1284 allows for estimation of +/− 4.6% of the proportions of the variables of interest after accounting for potential cluster effect from the three facility groupings. Compared to eligible non-participants, participants were less likely to be of Aboriginal or Torres Strait Islander origin (9.1% vs 3.4%, *p* < .0001) and less likely to come from regional/remote towns (81.4% vs 75.6%).

**Table 1:** Client and community health service characteristics, 2009–10, NSW, Australia (N = 1284)

**Characteristic**	**Participants n (%)**
Service type	
Aged care	66 (5.1)
Allied health	300 (23.4)
Community child and family health^a^	203 (15.8)
Community nurses and other nursing services	434 (33.8)
Diabetes	106 (8.3)
Other^b^	175 (13.6)
Gender	
Female	831 (64.7)
Age	
<40	304 (23.7)
40-49	93 (7.2)
50-59	145 (11.3)
60+	742 (57.8)
SEIFA Index of Disadvantage^c^	
Lower	989 (77.0)
Higher	295 (23.0)
Client remoteness (ARIA)^d^	
Major cities	314 (24.5)
Regional/remote towns	970 (75.6)
Number of visits to service in prior 12 months^e^	
1	312 (24.4)
2-4	312 (24.4)
5-11	335 (26.2)
12+	321 (25.1)
Aboriginal and/or Torres Strait Islander^f^	
Yes	44 (3.4)
Marital status^g^	
Living with partner	752 (58.6)
Education^h^	
Some high school or less	361 (28.2)
Completed high school	612 (47.8)
Technical certificate or diploma	171 (13.4)
University or college degree, or higher	137 (10.7)
Employment	
Employed	263 (20.5)
Unemployed (or unable to work due to health reasons)	150 (11.7)
Retired	655 (51.0)
Other (e.g. student, home duties)	216 (16.8)
Number of conditions in the prior two months for which client needed to take medication or receive medical attention^i^	
0	192 (19.0)
1	336 (33.2)
2	196 (19.4)
3	134 (13.2)
4 or more	154 (15.2)

^a^Clients over 18 years of age (e.g. the parent of the child seeing the service).

^b^Other service types include: rehabilitation, chronic and complex care, women’s services, migrant services, renal/dialysis, and regional health service programs.

^c^2006 index of relative socio-economic advantage/disadvantage calculated using client postcodes, based on a continuum of advantage (high values, referring to the higher NSW half [>991]) and disadvantage (low values, referring to the lower NSW half [<=991]) [57].

^d^Access/Remoteness Index of Australia (ARIA)[58] and was calculated using client postcodes.

^e^Categories based on quartiles. 4 missing values.

^f^2 missing values.

^g^1 missing value.

^h^3 missing values.

^i^272 missing values.

### Behavioral risk prevalence

Eighty-eight per cent of clients reported at least one risk, with inadequate fruit or vegetable consumption the most prevalent (80.5%) and smoking the least (13.3%) (Table 2).

**Table 2:** **Weighted prevalence**^**a **^**of risk, preventive care delivery and acceptability, 2009–10, NSW, Australia**

**Variables**	**n (%)**
**Prevalence of risks** (N = 1284)	
Smoking	170 (13.3)
Fruit or vegetable under consumption (N = 1282)	1032 (80.5)
Alcohol overconsumption	285 (22.2)
Physical inactivity	363 (28.3)
Number of risks (N = 1282)	
0	159 (12.4)
1	572 (44.6)
2	396 (30.9)
3	136 (10.6)
4	20 (1.5)
**Prevalence of preventive care delivery**	
Assessment (N = 1284)	
Smoking	769 (59.9)
Fruit and vegetable under consumption	347 (27.0)
Alcohol overconsumption	626 (48.7)
Physical inactivity	549 (42.8)
All risks	207 (16.1)
Brief advice (for ‘at risk’ clients)	
Smoking (N = 170)	105 (61.7)
Fruit or vegetable under consumption (N = 1032)	207 (20.0)
Alcohol overconsumption (N = 285)	88 (30.8)
Physical inactivity (N = 363)	138 (38.0)
All applicable risks (N = 1123)	182 (16.2)
Offered to arrange referral (for ‘at risk’ clients)	
Smoking (to Quitline; N = 170)	8 (4.5)
Fruit or vegetable under consumption (to Get Healthy; N = 1032)	9 (0.9)
Alcohol overconsumption (to GP/AMS; N = 285)	4 (1.5)
Physical inactivity (to Get Healthy; N = 363)	4 (1.2)
All applicable risks (N = 1123)	7 (0.6)
Offered to send a summary of risks to GP/AMS (N = 1123)	171 (15.2)
Offered to arrange referral for all applicable risks, or offered to send a summary of risks to GP/AMS (N = 1123)	174 (15.5)
**Agreed preventive care acceptable**	
Assessment	
Smoking (N = 1284)	1223 (95.3)
Fruit and vegetable under consumption (N = 1282)	1134 (88.4)
Alcohol overconsumption (N = 1283)	1194 (93.1)
Physical inactivity (N = 1284)	1196 (93.1)
All risks (N = 1282)	1063 (82.9)
Brief advice (for ‘at risk’ clients)	
Smoking (N = 170)	151 (88.7)
Fruit or vegetable under consumption (N = 1032)	891 (86.3)
Alcohol overconsumption (N = 283)	252 (89.0)
Physical inactivity (N = 363)	323 (88.9)
All applicable risks (N = 1123)	932 (83.0)
Offered to have a referral arranged (for ‘at risk’ clients)	
Smoking (N = 170)	145 (85.3)
Fruit or vegetable under consumption (N = 1032)	814 (78.9)
Alcohol overconsumption (N = 283)	236 (83.3)
Physical inactivity (N = 363)	307 (84.5)
All applicable risks (N = 1123)	854 (76.0)

^a^Numbers may not add to total due to weighting.

### Prevalence of preventive care delivery

There was an inverse relationship between the prevalence of risk and the prevalence of preventive care, whereby care was most frequently provided for smoking (despite being the least prevalent risk) and least frequently for inadequate fruit or vegetable consumption (despite being most prevalent) (Table 2). Sixty per cent of all clients had their smoking status assessed, and 61.7% and 4.5% of smokers were provided brief advice and offered a referral respectively. Twenty-seven per cent of all clients had their fruit and vegetable consumption assessed, and 20.0% and 0.9% of ‘at risk’ clients were provided with brief advice and offered a referral respectively. Sixteen percent of clients were assessed for all four risks. Of those with at least one risk, 16.2% were provided with brief advice for all risks, 15.2% were offered to have a risk summary sent to their GP/AMS, and 0.6% were offered a specific referral for each risk.

### Client and community health service characteristics associated with preventive care delivery

#### Individual risks

Service type was the only variable found to be significantly associated with care in all the final regression models, with allied health services generally having significantly lower odds of care provision than most other service types for assessment and provision of advice for each risk (Table 3). Compared to allied health services, diabetes services had the highest relative odds of care for each risk.

**Table 3:** **Multi-variate analysis**^**a **^**regarding characteristics associated with assessment, and brief advice for individual risks, 2009–10, NSW, Australia**

**Variables included in the final regression models**^**b**^	**Assessment (N = 1284)**^**c**^		**Brief advice**		
	**OR (95% CI)**	***p***	**n (%)**	**OR (95% CI)**	***p***
**Smoking**					
**Service type**		<.001	N = 175		<.001
Aged care	1.8 (1.0 – 3.2)*		2 (1.1)	1.8 (0.1 – 32.7)	
Community child and family health^d^	4.4 (2.8 - 6.9)*		31 (17.7)	7.7 (2.5 - 23.9)*	
Community nurses and other nursing services	1.5 (1.1 – 2.1)*		55 (31.4)	3.2 (1.2 – 8.5)*	
Diabetes	14.5 (6.5 – 32.6)*		13 (7.4)	68.6 (7.4- 636.2)*	
Other^e^	4.3 (2.6 - 7.0)*		20 (11.4)	14.0 (1.9 – 100.8)*	
Allied health	1.0		54 (30.9)	1.0	
**Gender**		.002			-
Male	1.6 (1.2 - 2.1)*		-	-	
Female	1.0		-	-	
**Fruit or vegetables**					
**Service type**		<.001	N = 1037		<.001
Aged care	2.2 (1.1 – 4.2)*		55 (5.3)	1.2 (0.4 – 2.9)	
Community child and family health^d^	1.1 (0.6 – 1.9)		169 (16.3)	0.9 (0.5 – 1.8)	
Community nurses and other nursing services	2.1 (1.4 – 3.2)*		348 (33.6)	1.5 (0.9 – 2.5)	
Diabetes	20.6 (10.9 – 39.1)*		81 (7.8)	13.7 (6.8- 27.3)*	
Other^e^	3.4 (2.1 – 5.6)*		144 (13.9)	3.8 (2.1 – 6.9)*	
Allied health	1.0		240 (23.1)	1.0	
**Aboriginality**		-	N = 1035		0.006
Yes, Aboriginal and/or Torres Strait Islander	-		39 (3.8)	3.1 (1.4 – 7.2)*	
No	-		996 (96.2)	1.0	
**Alcohol**					
**Service type**		<.001	N = 289		<.001
Aged care	1.6 (0.9 – 2.8)		14 (4.8)	0.4 (0.1 – 2.3)	
Community child and family health^d^	2.4 (1.4 - 4.0)*		25 (8.7)	0.6 (0.2 – 2.0)	
Community nurses and other nursing services	1.0 (0.7 – 1.4)		81 (28.0)	0.6 (0.2 – 1.3)	
Diabetes	11.6 (5.6 –24.4)*		26 (9.0)	10.6 (3.1- 36.3)*	
Other^e^	3.8 (2.4 – 6.0)*		58 (20.1)	1.1 (0.5 – 2.7)	
Allied health	1.0		85 (29.4)	1.0	
**Times client seen in last 12 months**		<.001			-
12+	0.7 (0.5 – 1.1)		-	-	
5-11	0.5 (0.3 - 0.7)*		-	-	
2-4	0.9 (0.6 – 1.3)		-	-	
1	1.0		-		
**SEIFA Index of Disadvantage**^**f**^		.010	-		
Higher NSW half [≤991]	1.6 (1.1 – 2.2)*		-	-	
Lower NSW half [>991]	1.0		-	-	
**Employment**		0.005	-		
Employed	0.9 (0.6 – 1.4)		-	-	
Unemployed (or unable to work due to health)	2.2 (1.4 – 3.4)*		-	-	
Other (e.g. Student, Home Duties)	0.8 (0.5 – 1.4)		-	-	
Retired	1.0		-	-	
**Physical activity**					
**Service type**		<.001	N = 361		<.001
Aged care	0.9 (0.5 – 1.7)		16 (4.4)	0.4 (0.1 – 1.7)	
Community child and family health^d^	0.4 (0.3- 0.7)*		82 (22.7)	0.5 (0.3 – 1.1)	
Community nurses and other nursing services	0.4 (0.2 – 0.5)*		92 (25.5)	0.6 (0.3 – 1.3)	
Diabetes	8.1 (4.0 –16.4)*		41 (11.4)	6.8 (2.4- 19.3)*	
Other^e^	2.1 (1.3 – 3.3)*		52 (14.4)	2.5 (1.1 – 5.7)*	
Allied health	1.0		78 (21.6)	1.0	
**Gender**		.002			-
Male	1.6 (1.2 - 2.2)*		-	-	
Female	1.0		-	-	
**Times client seen in last 12 months**		<.01			-
12+	2.0 (1.3 – 3.1)*		-	-	
5-11	1.2 (0.8 - 1.8)		-	-	
2-4	1.5 (1.0 – 2.2)		-	-	
1	1.0		-		

^a^Logistic regression analyses weighted and adjusted by facility groupings from the intervention trial [48].

^b^Final regression models which show only variables with significant independent associations with outcome after the modeling process.

^c^For N (%) for each variable included in the model refer to Table 1.

^d^Clients over 18 years of age (e.g. the parent of the child seeing the service).

^e^Other service types include: rehabilitation , chronic and complex care, women's services, migrant services, renal/dialysis, and regional health service programs.

^f^2006 index of relative socio-economic advantage/disadvantage calculated using client postcodes, based on a continuum of advantage (high values, referring to the higher NSW half [>991]) and disadvantage (low values, referring to the lower NSW half [<=991]) [57].

*Significant.

Client characteristics associated with assessment or brief advice varied according to the risk (Table 3). Males were more likely to be assessed for smoking and physical activity. Aboriginal clients were more likely to be provided brief advice regarding fruit or vegetable consumption. Compared to clients with one appointment (of which the majority were new clients), clients with between 5–11 prior service appointments in the last 12 months were less likely to have alcohol status assessed, and clients with 12 or more appointments were more likely to have physical activity assessed. Clients most socio-economically disadvantaged, and those unemployed (compared to retired clients) were more likely to have alcohol status assessed.

#### All risks

Service type was the only characteristic that remained significant in all three regression models for all three forms of preventive care. Compared to allied health services, diabetes and ‘other’ services were more likely to provide assessment, advice and referral/follow-up (Table 4).

**Table 4:** **Multi-variate analysis**^**a **^**regarding characteristics associated with assessment, brief advice, and offering referral/follow-up, for all risks combined, 2009–10, NSW, Australia**

**Variables included in the final regression model**	**Assessment (N = 1284)**^**b**^		**Brief advice (N = 1132)**			**Offer to arrange referral**^**c **^**(N = 1131)**		
	**OR (95% CI)**	***p***	**n (%)**	**OR (95% CI)**	***p***	**n (%)**	**OR (95% CI)**	***p***
**Service type**		<.001			<.001			<.001
Aged care	2.0 (0.9 – 4.4)		57 (5.0)	1.0 (0.4 – 3.0)		57 (5.0)	0.8 (0.3 – 2.2)	
Community child and family health^d^	0.8 (0.4 - 1.6)		183 (16.2)	1.1 (0.6 – 2.3)		183 (16.2)	0.8 (0.4 – 1.5)	
Community nurses and other nursing services	1.1 (0.6 – 1.8)		381 (33.7)	1.6 (0.9 – 2.7)		381 (33.7)	1.2 (0.7 – 2.1)	
Diabetes	17.4 (9.3 – 32.6)*		94 (8.3)	14.1 (7.2– 27.5)*		94 (8.3)	3.0 (1.5 – 5.8)*	
Other^e^	2.8 (1.6 - 5.0)*		154 (13.6)	2.7 (1.5 – 5.2)*		154 (13.6)	2.2 (1.2 – 4.0)*	
Allied health	1.0		263 (23.2)	1.0		262 (23.2)	1.0	

^a^Logistic regression analyses weighted and adjusted by facility groupings from the intervention trial [48].

^b^For N (%) for each variable included in the model refer to Table 1.

^c^Offered to arrange referral for all applicable risks, or asked for consent to send health habits summary to GP/AMS.

^d^Clients over 18 years of age (e.g. the parent of the child seeing the service).

^e^Other service types include: rehabilitation , chronic and complex care, women’s services, migrant services, renal/dialysis, and regional health service programs.

*Significant.

No interactions were found between variables that remained in the final regression for any of the models.

### Client acceptability of preventive care

Acceptability of assessment for the four individual risks ranged from 88.4% to 95.3%; for brief advice, 86.3% to 89.0%; and for arranging further support, from 78.9% to 85.3% (Table 2). The majority of clients found it acceptable for community health services to assess all risks (82.9%), and when ‘at risk’, for them to provide brief advice (83.0%) and to arrange further support (76.0%) for all risks.

## Discussion

The study findings suggest community health clinicians do not provide preventive care in a manner that is consistent with clinical guidelines. The prevalence of clinician assessment did not exceed 60% for any individual risk, and only 16% of clients were assessed for all four risks. Further, the form of preventive care most likely to result in a reduction of risk – referral/follow-up [14], was offered to less than 5% of clients for individual risks, and to less than 1% for all risks. Preventive care was preferentially provided to clients according to the type of behavioral risk and to the type of service attended. Strong client support existed for the provision of preventive care.

Comparison of the prevalence of preventive care found in this study with that previously reported is constrained by differences between studies in the definition of preventive care [34,42], the behavioral risks addressed [34,42,43], data collection methods [34-43], and the type of client [35,41-43]. The prevalence of smoking assessment has been reported in two previous medical record audit studies to range from 30% [36] to 71% [38], as compared to 59.9% in this study. One Australian study utilizing clinician self-report [34] has reported assessment of the four behavioral risks to occur for between 61.5% and 72.0% of clients, as compared to the 27.0% to 59.9% reported by clients in this study. The prevalence of assessment of all four risks combined, was lower in this study than that reported in an Australian clinician report-based study (16.1% vs 54.1%) [34].

With regard to the provision of brief advice, the prevalence in this study ranged from 20.0% for inadequate fruit and vegetable consumption to 61.7% for smoking, higher than that found in a medical records audit study (2% to 3% across the four risks) [43]. The prevalence of referral/follow-up found in this study (0.9% to 4.5% for individual risks and 0.6% for all risks) was generally lower than that found in clinician self-report studies regarding smoking (0% to 31.5%) [40,41], and multiple risks (86.3%) [34]. However these higher proportions are likely to be attributable to differences in the definition of referral/follow-up, whereby this could have included communicating the clients smoking plans and progress to the team [41] or brief advice and/or referral [34]. Overall, the provision of each of the three preventive care elements was delivered in a manner incongruent to the prevalence of the four behavioral risks (whereby despite being the least prevalent risk, smoking care was most frequently provided, and nutrition care was least frequently provided despite being the most prevalent risk), possibly partly attributable to the more well established smoking cessation guidelines [60].

The type of community health service attended was consistently found to be associated with preventive care delivery, with allied health services among those least likely to provide such care, and diabetes and ‘other’ services more likely to do so. Previous research has similarly found that services with a focus on chronic disease treatment [9,34,61,62] were more likely to provide preventive care [41,45], and that allied health professionals were the least likely to do so [45]. Such a finding is consistent with the findings of studies conducted in other clinical settings which suggest that when preventive care is provided, it occurs not from an opportunistic primary prevention perspective [13,14], but as an element of the diagnosis and treatment of risk-related conditions such as tobacco or obesity-related disease [63-73]. These findings confirm the need for additional strategies to support community health clinicians to adopt an opportunistic primary prevention approach to the provision of preventive care to all clients, and reinforce that practice change strategies are required to support policies regarding the delivery of such care. Furthermore, the findings suggest additional strategies are required to support services that traditionally do not deliver preventive care, and to support all clinicians to provide this for all clients, regardless of their demographic characteristics. In light of guidelines recommending all primary care clinicians- which includes allied health professionals- to provide preventive care across the four health risk behaviors [13] further professional development and practice redesign is required in order to address the current gap in care provision by such professionals.

Limited evidence has been reported describing the effectiveness of intervention strategies to increase the provision of any form of preventive care in community health settings [35,40,43]. Practice change theories [74-77] and evidence from practice change interventions in other clinical settings however suggest that a multi-strategic approach is most likely to increase clinician provision of preventive care on an opportunistic basis [18,78-83].

This study is one of few to involve a cross-sectional sample of clients from a large number of community health service facilities, a design that allows for a diverse range of clients and community health service types to be included. Secondly, the study is believed to be the first to comprehensively address separately the prevalence of three recommended forms of preventive care applied to four chronic disease behavioral risks in the community health setting. The study is one of few to have utilized client self-report [35,39], which has demonstrated greater accuracy than clinician self-report or medical records audit for reporting clinician performance regarding counseling behaviors [84]. While using a self-reported outcome possibly overestimated care provision [69,85,86], this re-enforces the low levels of preventive care delivery reported. The accuracy of outcome measures may have been affected by client recall over an extended period [84], however measurement via direct observation is not always feasible or ethical, and can be difficult and costly [84].

## Conclusions

The finding in this study that almost all community health clients report that the provision of preventive care is acceptable, is consistent with previous studies of clients from community health services [34,35,62] and other clinical settings [87], and provides a strong basis for clinicians to deliver this form of care. Such findings strengthen the need for strategies that facilitate clinician delivery of preventive care to be utilized if clinical guidelines are to be adhered to.

## Competing interest

The authors declare that they have no competing interests.

## Authors’ contributions

KM led the development of this manuscript. Authors MF, JK, and JW conceived the intervention concept. Authors MF, EC, JB, LW, JK, PW, JW, and SM secured grant funding from the National Health and Medical Research Council. SM provided clinical approval, leadership and liaison with community health district staff. CL provided statistical assistance regarding data analysis. All authors contributed to the research design and trial methodology and contributed to, read and approved the final version of this manuscript.

## Pre-publication history

The pre-publication history for this paper can be accessed here:

http://www.biomedcentral.com/1472-6963/13/167/prepub
